# Identification of distinct clinical phenotypes of cardiogenic shock using machine learning consensus clustering approach

**DOI:** 10.1186/s12872-023-03380-y

**Published:** 2023-08-29

**Authors:** Li Wang, Yufeng Zhang, Renqi Yao, Kai Chen, Qiumeng Xu, Renhong Huang, Zhiguo Mao, Yue Yu

**Affiliations:** 1 Department of Nephrology, Changzheng Hospital, Naval Medical University, Shanghai, China; 2 Department of Cardiothoracic Surgery, Changzheng Hospital, Naval Medical University, Shanghai, China; 3 Translational Medicine Research Center, Fourth Medical Center and Medical Innovation Research Division of the Chinese PLA General Hospital, Beijing, China; 4 Department of Burn Surgery, Changhai Hospital, Naval Medical University, Shanghai, China; 5 Research Unit of key techniques for treatment of burns and combined burns and trauma injury, Chinese Academy of Medical Sciences, Shanghai, China; 6 Department of Orthopedics, Changhai Hospital, Naval Medical University, Shanghai, China; 7 Department of Orthopaedics, Changzheng Hospital, Naval Medical University, Shanghai, China; 8 Department of General Surgery, Comprehensive Breast Health Center, Ruijin Hospital, Jiaotong University School of Medicine, Shanghai, China

**Keywords:** Acute kidney injury, Artificial intelligence, Cardiogenic shock, Cluster, Intensive care unit, Machine learning, Mortality, Phenotype

## Abstract

**Background:**

Cardiogenic shock (CS) is a complex state with many underlying causes and associated outcomes. It is still difficult to differentiate between various CS phenotypes. We investigated if the CS phenotypes with distinctive clinical profiles and prognoses might be found using the machine learning (ML) consensus clustering approach.

**Methods:**

The current study included patients who were diagnosed with CS at the time of admission from the electronic ICU (eICU) Collaborative Research Database. Among 21,925 patients with CS, an unsupervised ML consensus clustering analysis was conducted. The optimal number of clusters was identified by means of the consensus matrix (CM) heat map, cumulative distribution function (CDF), cluster-consensus plots, and the proportion of ambiguously clustered pairs (PAC) analysis. We calculated the standardized mean difference (SMD) of each variable and used the cutoff of ± 0.3 to identify each cluster’s key features. We examined the relationship between the phenotypes and several clinical endpoints utilizing logistic regression (LR) analysis.

**Results:**

The consensus cluster analysis identified two clusters (Cluster 1: *n* = 9,848; Cluster 2: *n* = 12,077). The key features of patients in Cluster 1, compared with Cluster 2, included: lower blood pressure, lower eGFR (estimated glomerular filtration rate), higher BUN (blood urea nitrogen), higher creatinine, lower albumin, higher potassium, lower bicarbonate, lower red blood cell (RBC), higher red blood cell distribution width (RDW), higher SOFA score, higher APS III score, and higher APACHE IV score on admission. The results of LR analysis showed that the Cluster 2 was associated with lower in-hospital mortality (odds ratio [OR]: 0.374; 95% confidence interval [CI]: 0.347–0.402; *P* < 0.001), ICU mortality (OR: 0.349; 95% CI: 0.318–0.382; *P* < 0.001), and the incidence of acute kidney injury (AKI) after admission (OR: 0.478; 95% CI: 0.452–0.505; *P* < 0.001).

**Conclusions:**

ML consensus clustering analysis synthesized the pattern of clinical and laboratory data to reveal distinct CS phenotypes with different clinical outcomes.

**Supplementary Information:**

The online version contains supplementary material available at 10.1186/s12872-023-03380-y.

## Introduction

Cardiogenic shock (CS), a state of circulatory failure, can occur due to acute ischaemic or non-ischaemic cardiac events, or from the progression of longstanding underlying heart disease [2–4]. Unfortunately, despite recent advances in pharmacological intervention or mechanical support, CS mortality remains unacceptably high and highly varied, with the 30-day mortality ranging from 50 to 90% [5]. The disparity in the mortality rates might imply CS patients are a heterogeneous population, and some phenotypes of CS are so different in clinical features and prognoses that they cannot be regarded as a whole population, both in clinical practice and research. Additionally, most attempts at staging CS have been based on expert opinions and consensus [6–9]. To avoid complexity, some of these classification systems only use few variables and depend on specific, although arbitrary, cutoffs, which may introduce bias and fail to capture the full variability of patient profiles [10]. Furthermore, the traditional logistic regression (LR) method has been used to develop most of these classifications, despite the fact that the predictors do not interact linearly and additively [11]. Therefore, a more accurate and granular classification of the CS spectrum is urgently needed to aid in the urgent and critical task of selecting proper management, including targeting the most appropriate candidates for advanced therapies.

Machine learning (ML) algorithms have become more commonly utilized in individualized medicine to support clinical decision-making as electronic medical records and artificial intelligence have advanced [12, 13]. Consensus clustering, an unsupervised ML approach, is used to find similarities and differences among numerous variables, and then allocate them into distinct phenotypes. Consensus clustering generates multiple clustering results by multiple iterations and merges these results to arrive at the final clustering result [14]. Additionally, consensus clustering can provide a visual display of multiple clustering results to help understand the clustering process and results, enhancing the interpretability of the algorithm. Recent studies have reported that ML consensus clustering approach may distinguish clinically distinct disease phenotypes such as cardiovascular diseases [9, 15].

Given the heterogeneity of patients with CS on admission [16], we aimed to identify clinically meaningful phenotypes of patients with CS using an unsupervised ML approach and to assess mortality risks among these distinct clusters.

## Methods

### Study design and data resource

We conducted a retrospective multi-center analysis using all the relevant data extracted from the electronic Intensive Care Unit (eICU) Collaborative Research database. The Database was a comprehensive ICU database for more than 200,000 admissions from over 200 hospitals across the USA between 2014 and 2015 [17]. We finished the “Protecting Human Research Participants” curriculum and obtained permission to access the dataset (authorization codes: 33,281,932). The establishment of the eICU database was approved by the Institutional Review Boards of the Massachusetts Institute of Technology (Cambridge, Massachusetts, USA). All the data were anonymized prior to research analyses by the eICU program, and hence the requirement for informed consent was waived. The study adhered to the ethical standards set forth in the 1964 Declaration of Helsinki and its later amendments.

### Patient selection

We included all critically ill patients with a primary diagnosis of CS using International Classification of Diseases, Ninth Revision (ICD-9) diagnosis codes from the eICU database (ICD-9 codes:758.51 and R57.0). Patients were excluded if they had: [1] the age of less than 18; [2] multiple ICU admissions; [3] a length of stay in the ICU less than 24 h; and [4] incomplete information about study outcomes (all-cause in-hospital mortality, all-cause ICU mortality, and the incidence of acute kidney injury [AKI] after admission). For patients with multiple admissions, we retained information only on the patient’s first admission to the ICU.

### Data extraction and processing

Demographic data, medical history, vital signs, laboratory data, scoring systems, treatment information, and others were retrieved from the eICU database using structured query language with PostgreSQL (version 13.6, www.postgresql.org). We only used data that was present within 24 h of ICU admission for clustering analysis since our aim was to phenotype CS patients based on available data at the time of ICU admission. If patients received vital signs measurement or laboratory tests more than once on the first day of admission, only the initial test results were considered for subsequent analyses.

The extracted variable included: [1] demographics: age, gender, ethnicity and body mass index (BMI); [2] ICU type: cardiac intensive care unit (CICU), cardiac surgery intensive care unit (CSICU), medical intensive care unit (MICU), surgery intensive care unit (SICU), cardiac care unit-cardiac trauma/surgical intensive care unit (CCU-CTICU), neuro intensive care unit (NICU), cardiac trauma intensive care unit (CTICU); [3] Medical history: myocardial infarction, coronary artery bypass grafting (CABG), percutaneous coronary intervention (PCI), pacemaker, congestive heart failure, cardiac arrhythmias, hypertension, peripheral vascular disease, chronic obstructive pulmonary disease (COPD), respiratory failure, stroke, neurologic disorders, diabetes, anemia, lymphoma, liver disease, peptic ulcer, metastatic cancer, rheumatoid arthritis, hypothyroidism, and acquired immunodeficiency syndrome (AIDS); [4] vital signs: systolic blood pressure (SBP), diastolic blood pressure (DBP), mean blood pressure (MBP), heart rate, respiratory rate, temperature, and oxygen saturation measured by pulse oximetry (SpO_2_); [5] laboratory findings: white blood cell (WBC) count, red blood cell (RBC) count, platelet count, red blood cell distribution width (RDW), blood urea nitrogen (BUN), creatinine, estimated glomerular filtration rate (eGFR), glucose, total protein, albumin, bilirubin, total calcium, potassium, sodium, chloride, and bicarbonate; The eGFR was calculated using the modification of diet in renal disease (MDRD) formula [18]; [6] prognostic scoring system: systemic inflammatory response syndrome (SIRS) score, Sequential Organ Failure Assessment (SOFA) score, acute physiology score III (APS III) and Acute Physiology and Chronic Health Evaluation IV (APACHE IV) sccore; [7] Treatment information: PCI, CABG, intraaortic balloon pump (IABP), mechanical ventilation, renal replacement treatment (RRT), and vasopressor use (dopamine, epinephrine, norepinephrine, or vasopressin). The definition of vasopressor use was at least one vasopressor was used during the first 24 h after admission.

### Endpoints

The study endpoints of our study included all-cause in-hospital mortality, all-cause ICU mortality, and the incidence of AKI after admission. KDIGO (Kidney Disease: Improving Global Outcomes) criteria were taken as the definition of AKI [19]. KDIGO criteria are as follows: increase in serum creatinine to ≥ 1.5 times baseline must have occurred within the prior 7 days, or a ≥ 0.3 mg/dl increase in serum creatinine occurred within 48 h, or urine volume < 0.5ml/kg/h for 6 h or more. The baseline serum creatinine was determined by using the minimum serum creatinine values available within the 7 days before admission. If the pre-admission serum creatinine was not available in the eICU database, the first serum creatinine measured at admission was used as the baseline serum creatinine.

### Management of missing data

Variables with more than 20% missing values were excluded since large amounts of missing data might cause bias. Correspondingly, for variables with fewer than 20% missing values, multivariable imputation was applied, which was based on 5 replications and a chained equation approach method. Additionally, the extreme values were not omitted and treated as missing data for imputation [20].

### Cluster analysis

We applied an unsupervised ML approach to consensus clustering to identify clinical phenotypes of ICU patients with CS. To prevent producing an excessive number of clusters that would not be clinically helpful, we employed a pre-specified subsampling parameter of 80% with 100 iterations and assigned the number of potential clusters (k) to vary from 2 to 8 in sequence with the K-means clustering algorithm. The optimal number of clusters was determined by cumulative distribution function (CDF) plot, delta area plot, consensus matrix (CM) heat map, cluster-consensus plot in the within-cluster consensus scores, and the proportion of ambiguously clustered pairs (PAC) analysis [21]. Pairwise consensus values, defined as ‘the proportion of clustering runs in which two items are grouped together’, are calculated and stored in a CM for each k. Then for each k, a final agglomerative hierarchical consensus clustering using distance of 1 − consensus values is completed and pruned to k groups, which are called consensus clusters. The within-cluster consensus score, ranging from 0 to 1, is defined as the average consensus value for all pairs of individuals within the same cluster [22]. A value closer to 1 indicates better cluster stability [22]. PAC is calculated as the proportion of all sample pairs with consensus values falling within the predetermined boundaries [21, 22]. A value closer to zero indicates better cluster stability [21].

### Statistical analysis

After we identified the clusters of CS patients, we performed analyses to test the differences among the clusters. Data were presented as mean ± standardized differences (SD) and compared between groups using a Student’s *t* test if the measurement data were normally distributed and the variance was homogeneous. If the requirements were not satisfied, data were expressed as median interquartile range (IQR), and the Kruskal Wallis rank test was used for comparisons between groups. Numeration data were reported as absolute numbers and percentages, with statistical analysis using Pearson’s χ2 test or Fisher’s exact test as appropriate.

We determined the clusters’ key features using an absolute standardized mean difference (SMD) of > 0.3 in reference to Thongprayoon’s studies [23–25]. We then compared outcomes among the identified clusters. We assessed the association of clusters with CS and in-hospital mortality, ICU mortality, and the incidence of AKI after admission using the LR model. Cluster 1 is taken as the reference group in the further analysis. The extracted variables were not incorporated into the LR analysis because these characteristics were used to identify clusters through unsupervised ML. We performed all analyses using R, version 4.0.5 (RStudio, Inc., Boston, MA, USA; http://www.rstudio.com/), with the package of Consensus ClusterPlus (version 1.54.0) for consensus clustering analysis [22].

## Result

### Identification of the optimal number of clusters

**Fig. 1:**
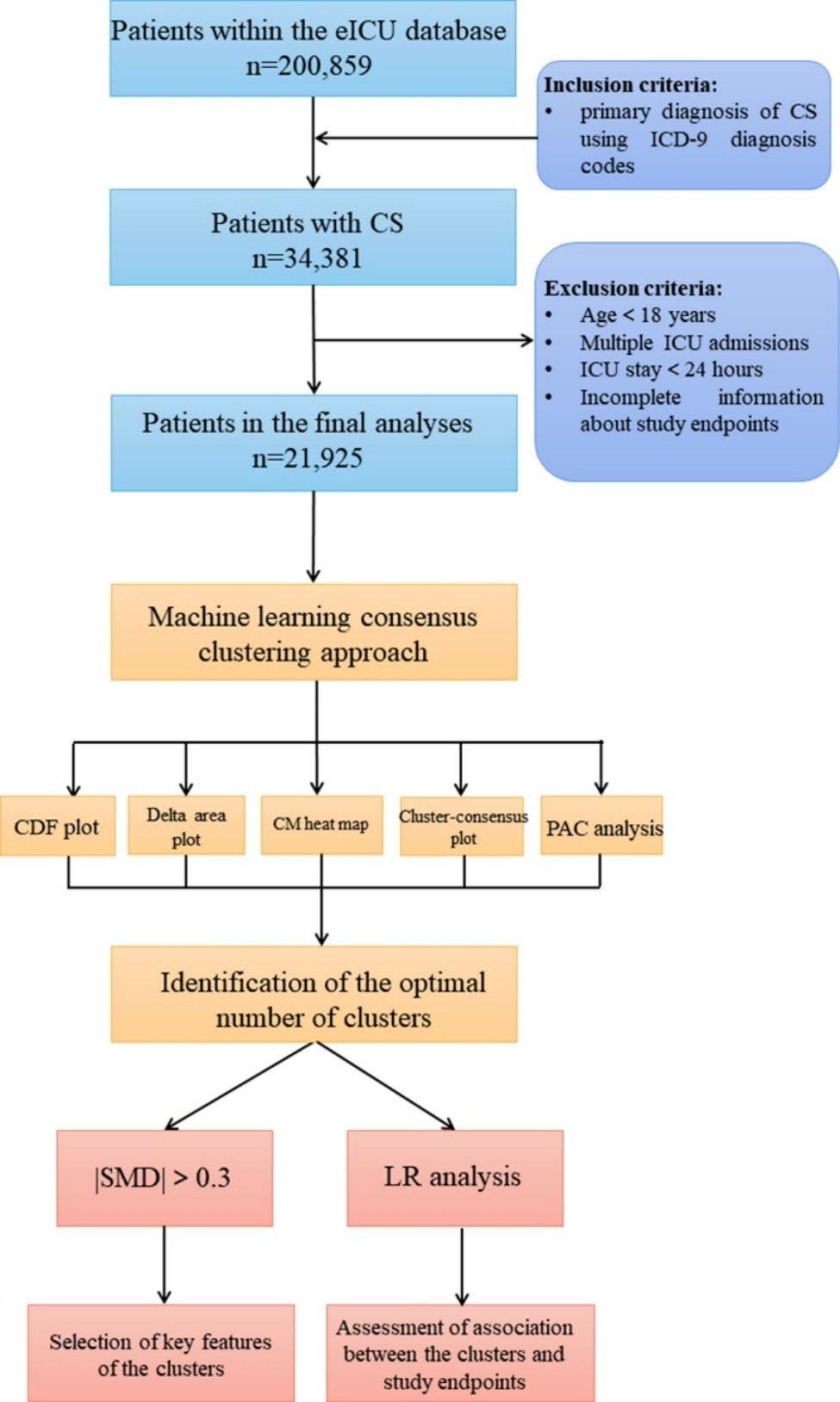
Flow diagram of patient inclusion and overview of the statistical analysis. Abbreviation: *CS: cardiogenic shock; ML: machine learning; eICU: electronic Intensive Care Unit; ICD-9: International Classification of Diseases, Ninth Revision; CDF: cumulative distribution function; CM: consensus matrix; PAC: proportion of ambiguously clustered pairs; SMD, standardized mean differences; LR: logistic regression*

**Fig. 2:**
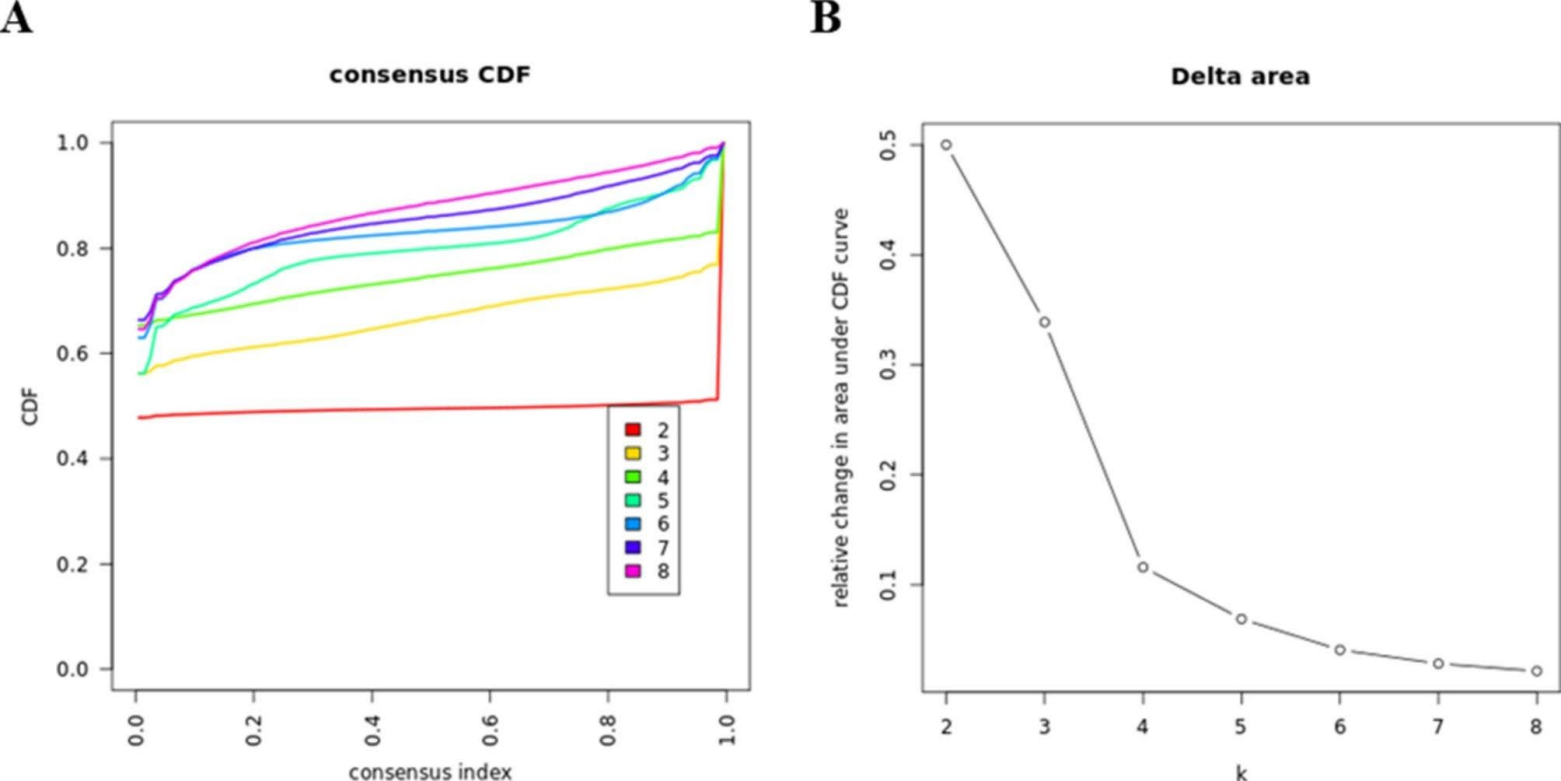
**(A)** CDF plot; **(B)** Delta area plot. Abbreviation: *CDF: cumulative distribution function*

Out of 34,682 ICU admissions, 21,925 patients with the diagnosis of CS were enrolled in the final cohort (Fig. 1). The CDF plot shows each cluster’s consensus distributions (Fig. 2A). The delta area plot displays the relative change in the area under the CDF curve, and the largest changes in area occurred between k = 2 and k = 5 (Fig. 2B). The CM heat map demonstrated that the ML algorithm identified cluster 2 and cluster 3 with clear boundaries, indicating good cluster stability over repeated iterations (Fig. S2A). As shown in the cluster-consensus plot, k = 2 and k = 3 had high stability given their high mean cluster consensus score (Fig. S2B). Additionally, favorable low PACs were demonstrated for 2 clusters (Fig. S1). Summarily, the ML consensus clustering approach from baseline characteristics on admission identified 2 clusters that best represented the data.

### Selection of Key features of the clusters

**Fig. 3:**
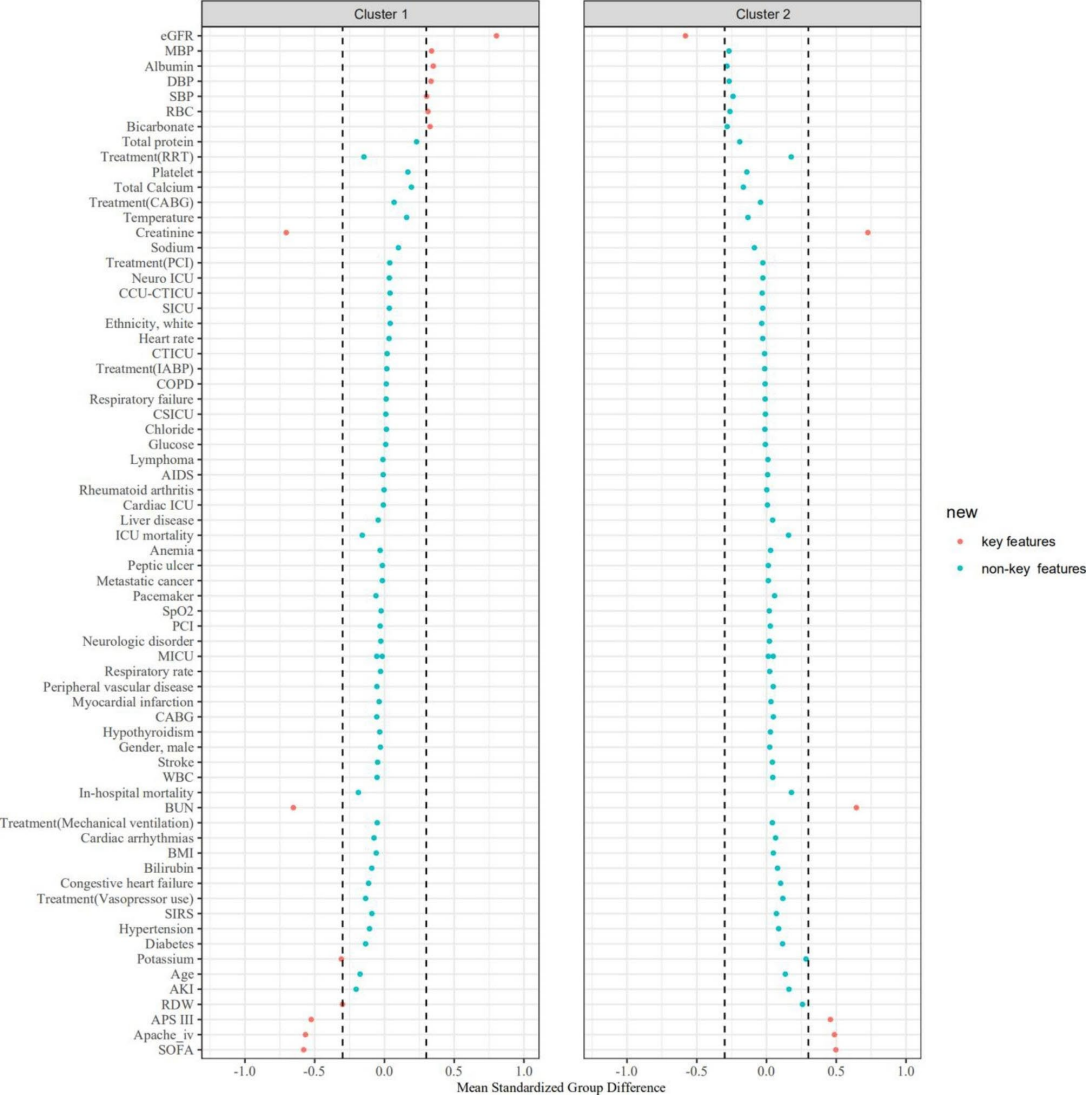
The SMD for each of baseline characteristics across clusters. Abbreviation: *SMD: standardized mean differences; BMI: body mass index; CICU: cardiac cardiac intensive care unit; CSICU: cardiac surgery intensive care unit; MICU: medical intensive care unit; SICU: surgery intensive care unit; CCU-CTICU: cardiac care unit-cardiac trauma/surgical intensive care unit; NICU: neuro intensive care unit; CTICU: cardiac trauma intensive care unit; CABG: coronary artery bypass grafting; PCI: percutaneous coronary intervention; COPD: chronic obstructive pulmonary disease; AIDS: acquired immunodeficiency syndrome; SBP: systolic blood pressure; DBP: diastolic blood pressure; MBP: mean blood pressure; SpO*_*2*_: *oxygen saturation measured by pulse oximetry; WBC: white blood cell; RBC: red blood cell; RDW: red blood cell distribution width; BUN: blood urea nitrogen; eGFR: estimated glomerular filtration rate; SIRS: systemic inflammatory response syndrome; SOFA: Sequential Organ Failure Assessment; APS III: acute physiology score III, APACHE IV: Acute Physiology and Chronic Health Evaluation IV; IABP: intraaortic balloon pump; RRT: renal replacement treatment*

Figure S3 shows missing rate for clinical and laboratory variables extracted from the database. Cluster 1 had 9,848 (44.9%) patients, while Cluster 2 had 12,077(55.1%) patients. As shown in Table 1, the clinical characteristics of the two identified clusters in the CS cohort were significantly different. Ages at presentation were 70 (59–80) years for Cluster 1 cohort, and 65 (63–76) years for Cluster 2, whereas male sex represented 53.8% and 51.2% of patients in these two cohorts, respectively. On the basis of the |SMD|>0.3, the key features of patients in Cluster 1, compared with Cluster 2, included: lower SBP, lower MBP, lower DBP, lower eGFR, higher BUN, higher creatinine, lower albumin, higher potassium, lower bicarbonate, lower RBC count, higher RDW, higher SOFA, higher APS III score, and higher APACHE IV score (Fig. 3 **and** Table 2).

**Table 1:** Baseline characteristics of the clusters

Characteristics	Total	Cluster 1 (*n* = 9848)	Cluster 2 (*n* = 12,077)	*P* value
**Demographics**				
Age, year	67 (56, 78)	70 (59, 80)	65 (53, 76)	< 0.001
Gender, male, %	11,471 (52.3)	5293 (53.8)	6178 (51.2)	< 0.001
Ethnicity, white, %	17,033 (77.7)	7482 (76.0)	9551 (79.1)	< 0.001
BMI, kg/m^2^	27.1 (23.0, 32.5)	27.4 (23.3, 33.1)	26.8 (22.8, 31.9)	< 0.001
**ICU type, %**				< 0.001
CICU	13,246 (60.4)	6026 (61.2)	7220 (59.8)	
Others	8679 (39.6)	3822 (38.8)	4857 (40.2)	
**Past history, %**	462 (2.1)	182 (1.9)	280 (2.3)	
Myocardial infarction	2130 (9.7)	1070 (10.9)	1060 (8.8)	< 0.001
CABG	1411 (6.4)	774 (7.9)	637 (5.3)	< 0.001
PCI	1397 (6.4)	704 (7.2)	693 (5.7)	< 0.001
Pacemaker	871 (4.0)	518 (5.3)	353 (2.9)	< 0.001
Congestive heart failure	4213 (19.2)	2354 (23.9)	1859 (15.4)	< 0.001
Cardiac arrhythmias	3238 (14.8)	1725 (17.5)	1513 (12.5)	< 0.001
Hypertension	11,536 (52.6)	5704 (57.9)	5832 (48.3)	< 0.001
Peripheral vascular disease	1203 (5.5)	667 (6.8)	536 (4.4)	< 0.001
COPD	3816 (17.4)	1666 (16.9)	2150 (17.8)	0.089
Respiratory failure	588 (2.7)	245 (2.5)	343 (2.8)	0.118
Stroke	2175 (9.9)	1125 (11.4)	1050 (8.7)	< 0.001
Neurologic disorder	4611 (21.0)	2175 (22.1)	2436 (20.2)	< 0.001
Diabetes	7233 (33.0)	3888 (39.5)	3345 (27.7)	< 0.001
Anemia	181 (0.8)	111 (1.1)	70 (0.6)	< 0.001
Lymphoma	184 (0.8)	93 (0.9)	91 (0.8)	0.143
Liver disease	336 (1.5)	211 (2.1)	125 (1.0)	< 0.001
Peptic ulcer	606 (2.8)	297 (3.0)	309 (2.6)	0.044
Metastatic cancer	741 (3.4)	360 (3.7)	381 (3.2)	0.045
Rheumatoid arthritis	489 (2.2)	224 (2.3)	265 (2.2)	0.723
Hypothyroidism	2397 (10.9)	1182 (12.0)	1215 (10.1)	< 0.001
AIDS	194 (0.9)	96 (1.0)	98 (0.8)	0.225
**Vital signs**				
SBP, mmHg	106 (92, 1)	100 (87, 1)	112 (98, 1)	< 0.001
DBP, mmHg	59 (50, 7)	54 (46, 6)	64 (55, 75)	< 0.001
MBP, mmHg	72 (62, 85)	67 (58, 78)	77 (67, 90)	< 0.001
Heat rate, beats/min	93 (79, 109)	92 (78, 108)	93 (80, 109)	< 0.001
Respiratory rate, beats/min	20 (16, 25)	20 (16, 25)	20 (16, 24)	< 0.001
Temperature, °C	36.8 (36.4, 37.2)	36.7 (36.3, 37.1)	36.8 (36.5, 37.3)	< 0.001
SpO_2_, %	98 (95, 100)	98 (95, 100)	98 (95, 100)	< 0.001
**Laboratory findings**				
WBC, 10^9^/L	12.1 (8.2, 17.2)	12.6 (8.3, 17.9)	11.8 (8.1, 16.6)	< 0.001
RBC, 10^9^/L	3.89 (3.3, 4.5)	3.59 (3.03, 4.2)	4.11 (3.6, 4.6)	< 0.001
Platelet, 10^9^/L	209 (149, 281)	192 (127, 264)	222 (165, 291)	< 0.001
RDW, %	15.1 (13.9, 16.8)	15.8 (14.5, 17.5)	14.6 (13.6, 16.0)	< 0.001
BUN, mg/dL	26 (17, 42)	41.1 (29, 57)	19 (13, 26)	< 0.001
Creatinine, mg/dL	1.32 (0.9, 2.2)	2.28 (1.5, 3.2)	0.99 (0.8, 1.3)	< 0.001
eGFR, mL/min/1.73m^2^	47.4 (25.2, 73.9)	24.6 (14.0, 39.5)	67 (48.5, 90.4)	< 0.001
Glucose, mg/dL	128 (104, 168)	128 (101, 171)	128 (105, 166)	0.003
Total protein, mg/dL	6.5 (5.7, 7.2)	6.2 (5.4, 6.9)	6.7 (5.9, 7.4)	< 0.001
Albumin, mg/dL	3 (2.5, 3.5)	2.7 (2.2, 3.2)	3.2 (2.7, 3.7)	< 0.001
Bilirubin, mg/dL	0.6 (0.4, 1.0)	0.7 (0.4, 1.0)	0.6 (0.4, 0.9)	< 0.001
Total Calcium, mmol/L	8.6 (8.1, 9.2)	8.4 (7.9, 9.0)	8.8 (8.3, 9.2)	< 0.001
Potassium, mmol/L	4.1 (3.7, 4.6)	4.4 (3.9, 5.0)	4 (3.6, 4.4)	< 0.001
Sodium, mmol/L	137 (133, 140)	136 (132, 140)	137 (134, 140)	< 0.001
Chloride, mmol/L	102 (97, 106)	101 (96, 106)	102 (97, 105)	0.005
Bicarbonate, mmol/L	24 (20, 27)	22 (18, 25)	25 (22, 28)	< 0.001
**Prognostic scoring system**				
SOFA	6 (4, 9)	8 (6, 11)	5 (3, 7)	< 0.001
SIRS	3 (2, 4)	3 (2, 4)	3 (2, 4)	< 0.001
APS III	52 (38, 70)	64 (51, 83)	43 (32, 56)	< 0.001
APACHE IV	66 (51, 84)	80 (66, 98)	56 (44, 70)	< 0.001
**Treatment information, %**				
PCI	142 (0.7)	37 (0.4)	105 (0.9)	< 0.001
CABG	235 (1.1)	46 (0.5)	189 (1.6)	< 0.001
IABP	296 (1.4)	114 (1.2)	182 (1.5)	0.030
Mechanical ventilation	10,682 (48.7)	5054 (51.3)	5628 (46.6)	< 0.001
RRT	824 (3.8)	697 (7.1)	127 (1.1)	< 0.001
Vasopressor use	6052 (27.6)	3329 (33.8)	2723 (22.6)	< 0.001

Values are presented as the means (standard deviations) or medians (interquartile ranges) for continuous variables, and categorical variables are presented as total numbers and percentages

*BMI: body mass index; CICU: cardiac cardiac intensive care unit; CABG: coronary artery bypass grafting; PCI: percutaneous coronary intervention; COPD: chronic obstructive pulmonary disease; AIDS: acquired immunodeficiency syndrome; SBP: systolic blood pressure; DBP: diastolic blood pressure; MBP: mean blood pressure; SpO*_*2*_: *oxygen saturation measured by pulse oximetry; WBC: white blood cell; RBC: red blood cell; RDW: red blood cell distribution width; BUN: blood urea nitrogen; eGFR: estimated glomerular filtration rate; SIRS: systemic inflammatory response syndrome; SOFA: Sequential Organ Failure Assessment; APS III: acute physiology score III, APACHE IV: Acute Physiology and Chronic Health Evaluation IV; IABP: intraaortic balloon pump; RRT: renal replacement treatment*

**Table 2:** Selection of key characteristics of the clusters

Characteristics	Cluster 1 (*n* = 9848)	Cluster 2 (*n* = 12,077)
**Blood pressure**		
SBP	100 (87, 1) low	112 (98, 1) high
DBP	54 (46, 6) low	64 (55, 75) high
MBP	67 (58, 78) low	77 (67, 90) high
**Kidney function**		
eGFR	24.6 (14.0, 39.5) low	67 (48.5, 90.4) high
BUN	41.1 (29, 57) high	19 (13, 26) low
Creatinine	2.28 (1.5, 3.2) high	0.99 (0.8, 1.3) low
**Liver function**		
Albumin	2.7 (2.2, 3.2) low	3.2 (2.7, 3.7) high
**RBC-associated indicators**		
RBC	3.59 (3.03, 4.2) low	4.11 (3.6, 4.6) high
RDW	15.8 (14.5, 17.5) high	14.6 (13.6, 16.0) low
**Electrolytes and acid-base compounds**		
Bicarbonate	22 (18, 25) low	25 (22, 28) high
Potassium	4.4 (3.9, 5.0) high	4 (3.6, 4.4) low
**Prognostic scoring system**		
SOFA	8 (6, 11) high	5 (3, 7) low
APS III	64 (51, 83) high	43 (32, 56) low
APACHE IV	80 (66, 98) high	56 (44, 70) low

*SBP: systolic blood pressure; DBP: diastolic blood pressure; MBP: mean blood pressure; BUN: blood urea nitrogen; eGFR: estimated glomerular filtration rate; RBC: red blood cell; RDW: red blood cell distribution width; SOFA: Sequential Organ Failure Assessment; APS III: acute physiology score III, APACHE IV: Acute Physiology and Chronic Health Evaluation IV.*

### Association of clusters with endpoints

**Fig. 4:**
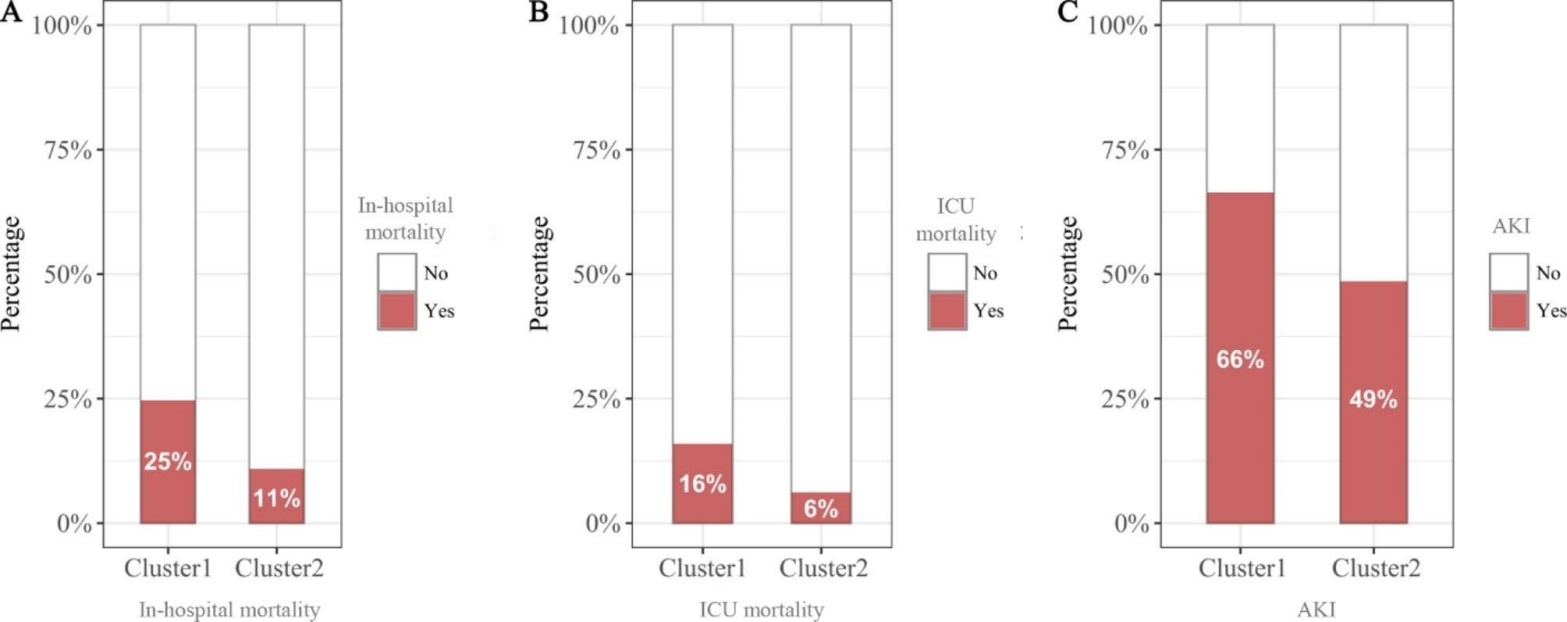
**(A)** In-hospital mortality, **(B)** ICU mortality, and **(C)** Incidence of AKI after admission among different clusters. Abbreviation: *AKI: acute kidney injury; ICU:intensive care unit*

**Fig. 5:**
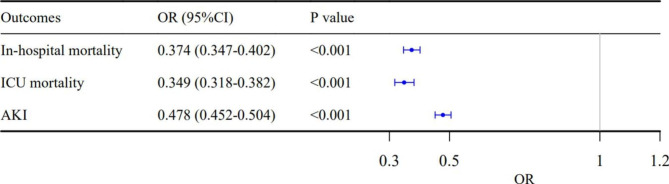
The forest plot of OR (95% CI) for **(A)** In-hospital mortality, **(B)** ICU mortality, and **(C)** Incidence of AKI after admission. Abbreviation: *OR: odds ratio; CI: confidence interval; AKI: acute kidney injury; ICU:intensive care unit*

The in-hospital mortality, ICU mortality, and incidence of AKI for Cluster 1 patients were 25%, 16%, and 66% respectively, and were 11%, 6%, and 49% for Cluster 2 patients, respectively (*P* < 0.001 for all) (Fig. 4 and Table S1). The results of LR analysis showed that the cluster 2 was associated with lower in-hospital mortality (odds ratio [OR]: 0.374; 95% confidence interval [CI]: 0.347–0.402; *P* < 0.001), ICU mortality (OR: 0.349; 95% CI: 0.318–0.382; *P* < 0.001), and incidence of AKI after admission (OR: 0.478; 95% CI: 0.452–0.505; *P* < 0.001) (Fig. 5).

## Discussion

### Major findings

The unsupervised ML consensus clustering approach provides the ability to more efficiently analyze, identify, and classify phenotypes of patients on admission based on large amounts of data. In this study, two distinct clusters of critically ill patients with CS were determined by applying the consensus clustering algorithm. Blood pressure (SBP, MBP, and DBP), kidney function (creatinine, BUN, and eGFR), electrolytes and acid-base compounds (potassium and bicarbonate), liver function (albumin), RBC-associated indicators (RBC count and RDW), and some scoring systems (SOFA, APS III, and APACHE IV) were the key features used to differentiate the phenotypes of CS. In addition, these two clusters were also associated with multiple study endpoints including in-hospital mortality, ICU mortality, and the incidence of AKI after admission. A more accurate and granular classification could deepen our understanding of CS pathophysiology, be introduced into clinical practice as a risk assessment tool, and provide participant selection information for clinical trials.

### Relation to other works

Several prognostic classifications or risk stratifications of CS have been reported. For example, with regard to hemodynamic phenotypes of CS, patients are generally classified into 4 phenotypes based on cardiac output (i.e., insufficient [cold] versus sufficient [warm]) and volume status (i.e., overloaded [wet] versus euvolemic [dry]) which reflect tissue perfusion and congestion, respectively [26, 27]. This classic “cold and wet” profile is the most frequent CS phenotype, accounting for nearly two-thirds of patients with MI-associated CS [28]. Based on 6 variables with a maximum of 9 points, there are three risk categories in the IABP-SHOCK II score [29]. Patients in the low, intermediate, and high risk categories have an in-hospital mortality risk of 20–30%, 40–60%, and 70–90%, respectively. The recently proposed Society of Cardiovascular Angiography and Interventions (SCAI) staging, describing stages of CS from A to E, provides discriminatory potential for morbidity and mortality [6]. It can be used to track the severity of shock over the course of a hospital stay. However, it was noted that some of these classification tools are based on expert consensus and theoretical considerations rather than on clinical evidence. To avoid complexity, some of these classifications contain only a few characteristics and depend on specific, although arbitrary, cutoff values that could result in bias and fail to capture the full variability of patient profiles. Additionally, some continuous variables in the classification were changed into categorized variable, which might cause a loss of information on between-subject variability. Furthermore, most of these classifications, using the traditional LR method, were developed assuming that the predictors interact in a linear and additive way, despite the reality that the interactions are often non-linear and multifactorial [11].

To address these limitations above, clustering analysis was used in this study to capture the natural structure of multivariate data without a priori knowledge and it has been applied extensively in medical science, for example, to identify clinical phenotypes [30]. It can also treat multiple variables independently and continuous variables as continuous. Zweck et al. [9] used machine learning, and identified 3 distinct CS phenotypes (“Noncongested” CS, “Cardiorenal” CS and “Cardiometabolic” CS), with specific and reproducible associations with mortality. However, their study and ours differ in terms of the study cohort, sample size, and statistical methods. Additionally, multiple endpoints (in-hospital mortality, all-cause ICU mortality, and the incidence of AKI) were set in our study. AKI, which is reflected by a rise in serum creatinine and a potential reduction in urinary output, may indicate renal hypoperfusion in the setting of CS and is associated with poor outcomes [31]. In the current study, 2 phenotypes were identified. Compared with those in Cluster 2, patients in Cluster 1 had worse hemodynamic and metabolic parameters, lower scoring systems, and worse clinical outcomes, which indicated they were more likely to suffer from multisystem organ failure [4, 29].

Through calculating the SMD of each variable, we determined that SBP, MBP, DBP, eGFR, BUN, creatinine, albumin, potassium, bicarbonate, RBC, RDW, SOFA, APS III, and APACHE IV were the key features between clusters. Some of these indicators have been found to be associated with risk of mortality in CS. A creatinine of greater than 1.33 had significantly higher mortality in the Intra-aortic Balloon Pump in CS (IABP-SHOCK II) trial [32]. Serum bicarbonate, especially when evaluated in the early-stage course of CS patients, could offer information regarding prognosis. Wigger et al. [33] found that serum bicarbonate decreased prior to significant elevation of lactate. A low bicarbonate level shows the better ability to predict 30-day mortality than the highest recorded lactate level. One recent study has reported that higher RDW is associated with an increased risk of all-cause mortality in critically ill patients with CS [34]. There is mounting evidence that the development of SIRS plays an important role in the pathogenesis of CS. Pierce et al. [35] found that inflammatory cytokines might cause an increase in RDW by affecting iron metabolism and inhibited bone marrow. Additionally, CS can cause activation of the renin-angiotensin system, which leads to an increase in RDW with erythropoiesis [36]. Multiple scoring systems derived from the ICU population have been proposed to predict clinical outcomes in CS. A small study comparing the APACHE-II, APACHE-III, SAPS-II, and SOFA scoring systems in CS reported that APACHE-III and SAPS-II had the best mortality discrimination [10]. The latest version of APACHE-IV is calculated based on 129 variables derived within the first 24 h of ICU admission, which was assessed from over 110,588 patients admitted to more than 104 ICUs across the USA [37, 38]. The application of the APACHE IV score is limited due to its complexity. However, as data science advances, the complexity of these scores could be overcome by electronic recording techniques and computing power.

### Clinical implications

The strengths of our study include innovative findings via an unsupervised ML consensus clustering approach derived from a large sample size consisting of a multi-center population of ICU patients with CS covering a broad spectrum of etiologies. The identified clusters of CS may be used by clinicians in the ICU to quickly assess patients with CS, as the key features identified in this study are rapid, easy, and inexpensive laboratory tests. These clusters may enhance clinical trials by developing treatment strategies tailored to a shock phenotype instead of aiming for a one-size-fits-all solution, thereby paving the way for more individualized health care. This new classification system of different shock states will also help to make different trials of CS better comparable and may also trigger new randomized trials on the pre-shock state.

### Limitations

There were several limitations to our current study. First, due to the retrospective nature of this study, future studies may collect comprehensive data in a prospective manner and allow for enhanced, even more nuanced examination of the CS phenotypes. Second, in the eICU database, values for some important variables, including lactate, brain natriuretic peptide, and some advanced hemodynamic monitoring parameters, were documented incompletely and not included for currentanalysis. Third, restricted by the eICU database, the etiology of CS has not been identified accurately. Future studies should attempt to conduct subgroup analyses based on different causes of CS. Fourth, consensus clustering was performed on hospital admission and did not include data before or during hospitalization, which could affect hospitalization-related outcomes. Lastly, our classification tool only enrolled the variable of CS at the early stage, and cannot evaluate the severity and progression of CS dynamically. Therefore, in the future, we will study the association between ML-derived phenotypes and endpoints within individual SCAI stages, whose aim is to characterize disease severity as it evolves over the course of a hospital stay.

## Conclusions

ML consensus clustering analysis identified distinct clusters of hospitalized CS. Blood pressure (SBP, MBP, and DBP), kidney function (creatinine, BUN, and eGFR), electrolytes (potassium and bicarbonate), liver function (total protein and albumin), RBC-associated indicators (RBC count and RDW), and some scoring systems (SOFA, APS III, and APACHE IV) were the key features used to differentiate the phenotypes of CS upon admission. Furthermore, the distinct phenotypes of CS have differing in-hospital mortality, ICU mortality, and the incidence of AKI after admission. Accordingly, these findings may help with risk classification and the design of treatment algorithms tailored to each phenotype of CS, as well as inform participant selection in future clinical trials.

### Electronic supplementary material

Below is the link to the electronic supplementary material.

Additional File 1: figures S1-S3 and table S1

## Data Availability

The datasets used and/or analysed during the current study are available from the corresponding author on reasonable request.

## Abbreviations

CS: Cardiogenic shock
ML: Machine learning
eICU: Electronic Intensive Care Unit
ICD-9: International Classification of Diseases Ninth Revision
BMI: Body mass index
CICU: Cardiac cardiac intensive care unit
CSICU: Cardiac surgery intensive care unit
MICU: Medical intensive care unit
SICU: Surgery intensive care unit
CCU-CTICU: Cardiac care unit-cardiac trauma/surgical intensive care unit
NICU: Neuro intensive care unit
CTICU: Cardiac trauma intensive care unit
CABG: Coronary artery bypass grafting
PCI: Percutaneous coronary intervention
COPD: Chronic obstructive pulmonary disease
AIDS: Acquired immunodeficiency syndrome
SBP: Systolic blood pressure
DBP: Diastolic blood pressure
MBP: Mean blood pressure
SpO_2_: Oxygen saturation measured by pulse oximetry
WBC: White blood cell
RBC: Red blood cell
RDW: Red blood cell distribution width
BUN: Blood urea nitrogen
eGFR: Estimated glomerular filtration rate
MDRD: Modification of diet in renal disease system
SIRS: Systemic inflammatory response syndrome
SOFA: Sequential Organ Failure Assessment
APS III: Acute physiology score III
APACHE IV: Acute Physiology and Chronic Health Evaluation IV
IABP: Intraaortic balloon pump
RRT: Renal replacement treatment
AKI: Acute kidney injury
KDIGO: Kidney Disease:Improving Global Outcomes
CDF: Cumulative distribution function
CM: Consensus matrix
PAC: Proportion of ambiguously clustered pairs
SD: Standardized differences
IQR: Interquartile range
SMD: Standardized mean differences
LR: Logistic regression
OR: Odds ratio
CI: Confidence interval
SCAI: Society of Cardiovascular Angiography and Interventions
