# Supplementary material for: Identification of distinct clinical phenotypes of cardiogenic shock using machine learning consensus clustering approach
Source: BMC Cardiovasc Disord. 2023 Aug 29;23:426. doi: 10.1186/s12872-023-03380-y (PMC10466857; doi:10.1186/s12872-023-03380-y)
Supplement: Supplementary file 1 — Additional File 1: figures S1-S3 and table S1 [file 12872_2023_3380_MOESM1_ESM.docx]

**Table S1. Outcomes of the clusters**

| Outcomes | **Total (*n*=21925)** | **Cluster 1 (*n*=9848)** | **Cluster 2 (*n*=12077)** | **P value** |
| --- | --- | --- | --- | --- |
| Hospital mortality, % | 17.1 (3744/21925) | 24.7 (2428/9848) | 10.9 (1316/12077) | <0.001 |
| ICU mortality, % | 10.6 (2318/21925) | 16.0 (1569/9848) | 6.2 (749/12077) | <0.001 |
| AKI, % | 56.6 (12400/21925) | 66.4 (6538/9848) | 48.5 (5862/12077) | <0.001 |

*AKI: acute kidney injury*

**
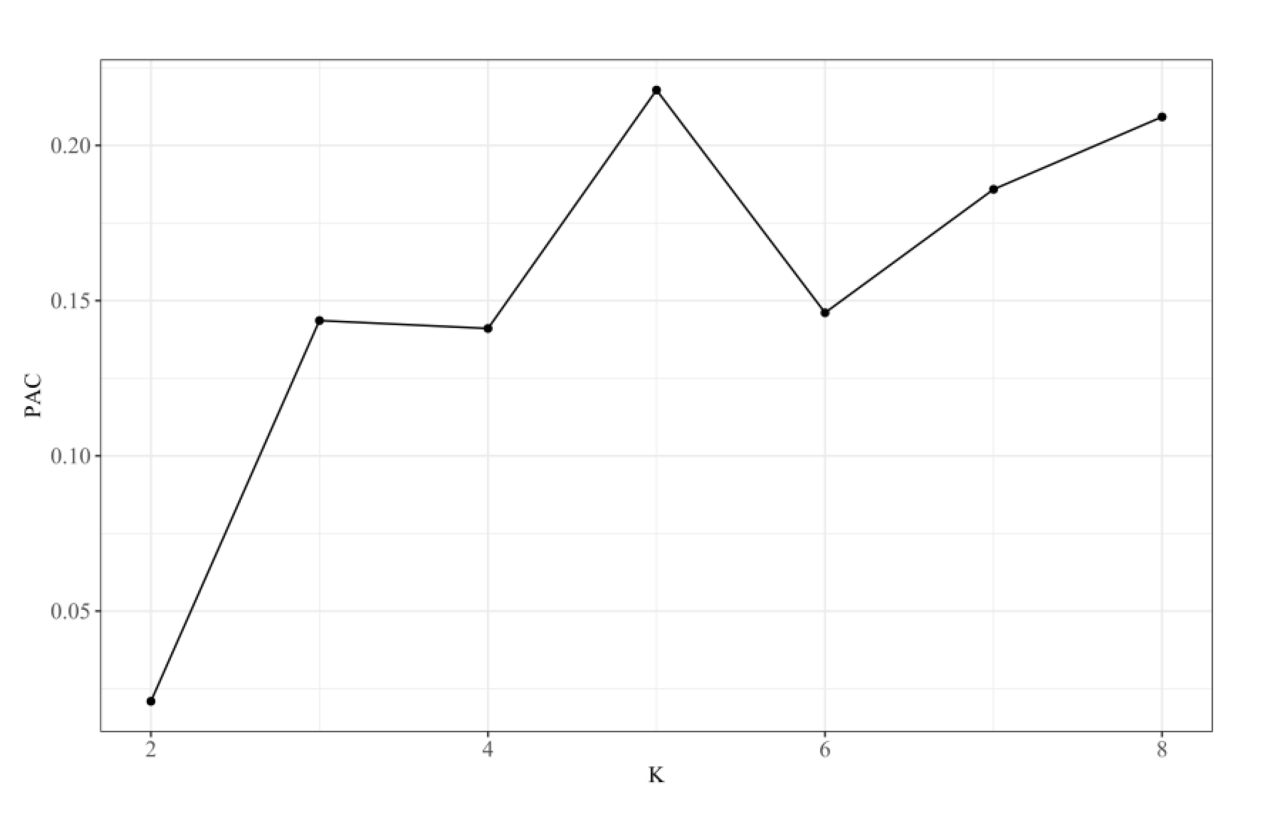
Figure S1**

**Figure S1 legend**

**Figure S1.** PAC analysis. PAC is calculated as the proportion of all sample pairs with consensus values falling within the predetermined boundaries. *PAC: proportion of ambiguously clustered pairs.*

**Figure S2**


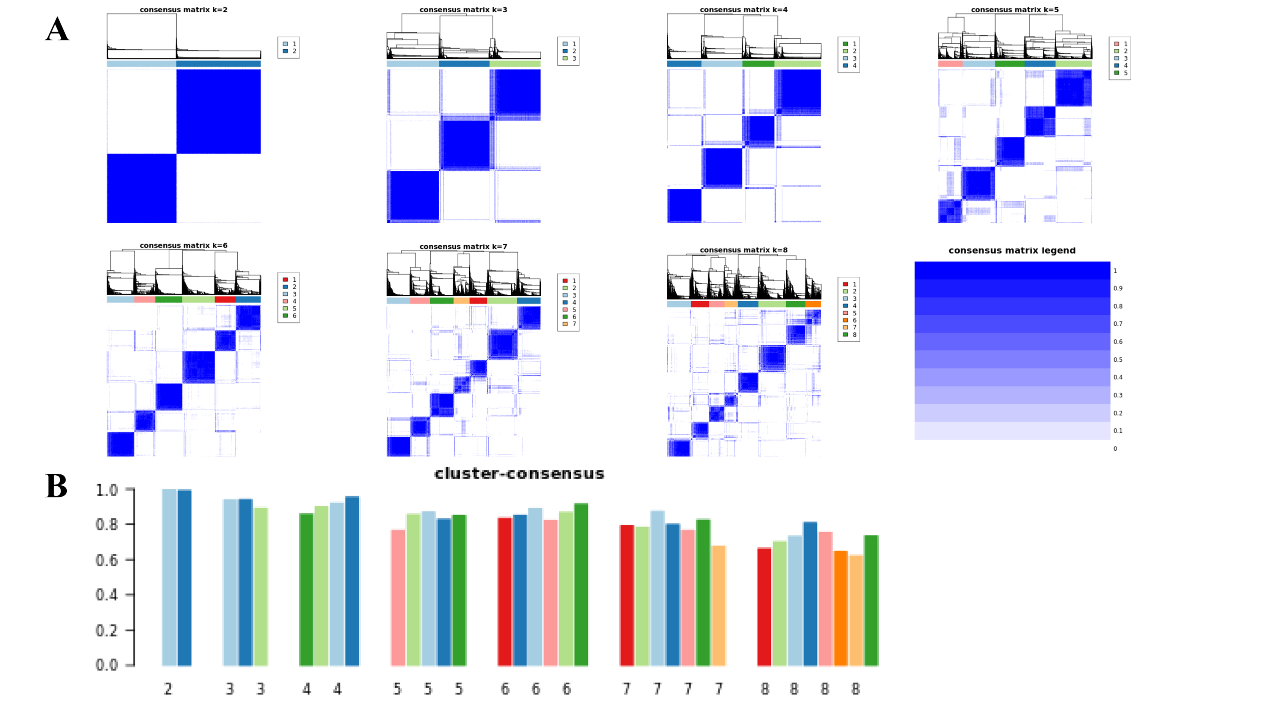


**Fig. S2.** (A) CM heat map; (B) Cluster-consensus plot. Abbreviation: *CM: consensus matrix.*

**Figure S3**


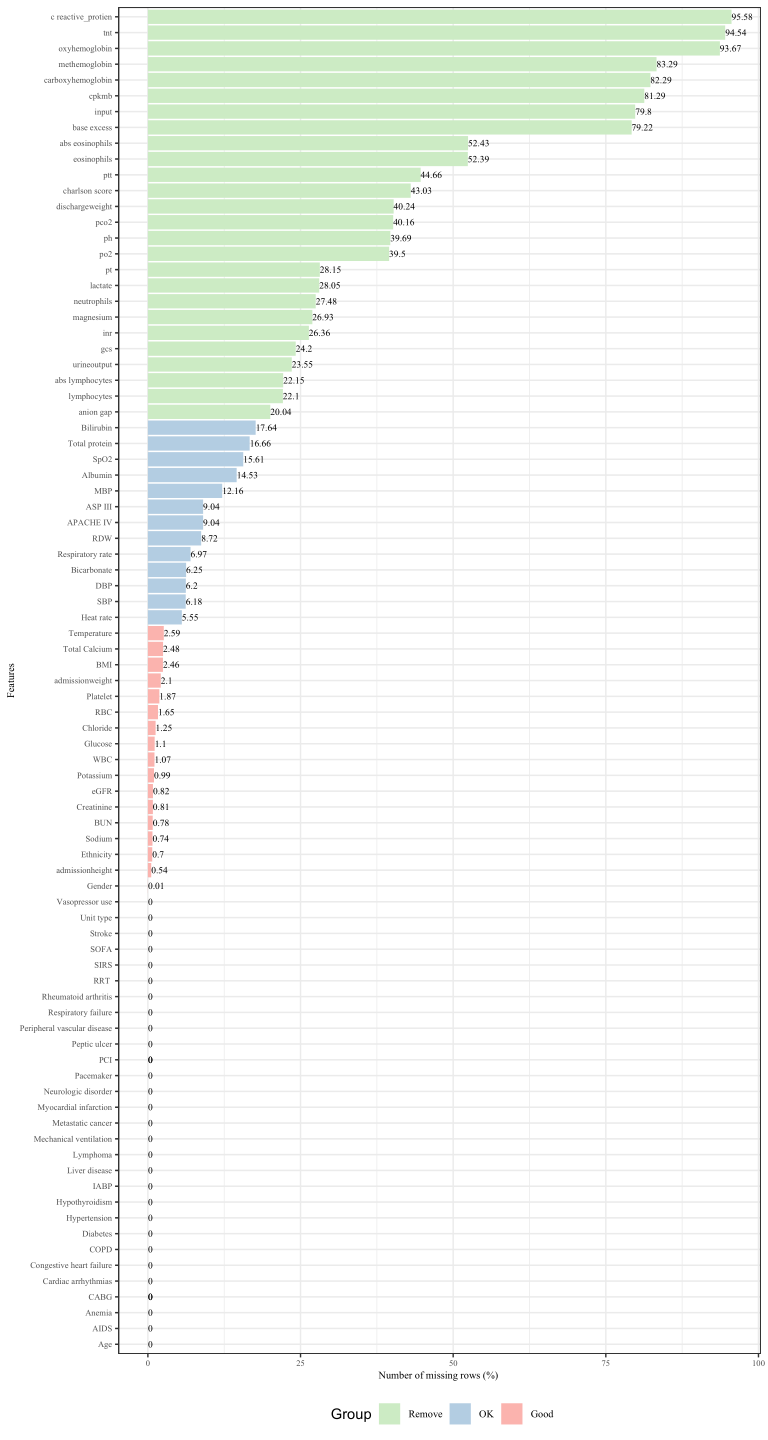


**Fig. S3***.* Missing rate for clinical and laboratory variables extracted from the database. *BMI: body mass index;CABG: coronary artery bypass grafting; PCI: percutaneous coronary intervention; COPD: chronic obstructive pulmonary disease; AIDS: acquired immunodeficiency syndrome; SBP: systolic blood pressure; DBP: diastolic blood pressure; MBP: mean blood pressure; SpO_2_: oxygen saturation measured by pulse oximetry; WBC: white blood cell; RBC: red blood cell; RDW: red blood cell distribution width; BUN: blood urea nitrogen; eGFR: estimated glomerular filtration rate; SIRS: systemic inflammatory response syndrome; SOFA: Sequential Organ Failure Assessment; APS III: acute physiology score III, APACHE IV: Acute Physiology and Chronic Health Evaluation IV; IABP: intraaortic balloon pump; RRT: renal replacement treatment.*
